# Identification of Potential Diagnostic Gene Targets for Pediatric Sepsis Based on Bioinformatics and Machine Learning

**DOI:** 10.3389/fped.2021.576585

**Published:** 2021-03-04

**Authors:** Ying Qiao, Bo Zhang, Ying Liu

**Affiliations:** ^1^Department of Pediatrics, Tianjin Union Medical Center, Tianjin, China; ^2^Tianjin Key Laboratory of Cellular and Molecular Immunology, Department of Immunology, School of Basic Medical Sciences, Tianjin Medical University, Tianjin, China

**Keywords:** sepsis, pediatric sepsis, GO enrichment analysis, KEGG enrichment analysis, logistic regression model, biomarker

## Abstract

**Purpose:** To develop a comprehensive differential expression gene profile as well as a prediction model based on the expression analysis of pediatric sepsis specimens.

**Methods:** In this study, compared with control specimens, a total of 708 differentially expressed genes in pediatric sepsis (case–control at a ratio of 1:3) were identified, including 507 up-regulated and 201 down-regulated ones. The Gene Ontology (GO) and Kyoto Encyclopedia of Genes and Genomes (KEGG) enrichment analysis of differentially expressed genes indicated the close interaction between neutrophil activation, neutrophil degranulation, hematopoietic cell lineage, *Staphylococcus aureus* infection, and periodontitis. Meanwhile, the results also suggested a significant difference for 16 kinds of immune cell compositions between two sample sets. The two potential selected biomarkers (MMP and MPO) had been validated in septic children patients by the ELISA method.

**Conclusion:** This study identified two potential hub gene biomarkers and established a differentially expressed genes-based prediction model for pediatric sepsis, which provided a valuable reference for future clinical research.

## Introduction

Sepsis is a life-threatening organ dysfunction initiated by an imbalance in the systemic inflammatory response to infection (1). Over the past few decades, numerous medical studies have proposed several definitions for sepsis, such as septicemia, sepsis, toxemia, bacteremia, endotoxemia, and so on (2). Sepsis is characterized by a general pro-inflammatory cascade that causes extensive tissue damages, which includes severe clinical spectrums, such as septic shock as well as multiple organ failures (3). Sepsis can be initiated by bacteria, fungi, as well as viruses, without specific treatment (4). In this case, the diagnosis of sepsis is particularly difficult because these patients have multiple comorbidities and underlying diseases (5). Sepsis is the leading cause of child mortality worldwide, which is estimated as 60% for children under 5 (6). A US cohort study indicated a significant increase for the annual incidence of severe pediatric sepsis (7).

Over the last decades, the management for pediatric sepsis has improved gradually (8). The current therapies in clinic include resuscitation, prompt and appropriate antimicrobial therapy, accurate fluid balance, blood glucose, as well as source control (9). However, we still lack a specific molecular therapy for this condition, except for antimicrobial therapy. Numerous trials of potential biological agents targeting different mediators of sepsis have failed (2). Despite advances in intensive care and supportive technology, the mortality rate of sepsis in children still stay in a high position without going down (10). The current recommendation for identifying sepsis is the SOFA score, which refers to Sequential (Sepsis-Related) Organ Failure Assessment. SOFA is a simple system, which uses accessible parameters in daily clinical practice to identify dysfunction or failure of the key organs as a result of sepsis (11, 12). The European Medicines Agency has accepted that a change in the SOFA score of 2 or more is an acceptable surrogate marker for sepsis (13). Unfortunately, the criteria still cannot confirm or refute the diagnosis of sepsis completely given the complexity of the sepsis response. Moreover, sepsis is a time critical emergency, as the disease may progress rapidly to organ failure, shock, and death, which require a prompt recognition.

Based on inflated misdiagnosis rate and poor accuracy of diagnosis, pediatric sepsis has brought great difficulty to clinical treatment. To this end, a more comprehensive approach to predict pediatric sepsis based on the specific target gene differential expression is required. To address these issues, in this study, we used a combination of bioinformatics and machine learning to screen out the potential biomarkers in the pathogenesis of pediatric sepsis specimens and then constructed a diagnosis model. All of these promising outcomes enriched the diagnosis of the disease, which provide tremendous help for pediatric sepsis study.

## Materials and Methods

### Data Source

This study utilized the mRNA chip data in the GEO database, and the samples were from data sets numbered GSE26440, GSE26378, and GSE66099. GSE26440 included 98 whole blood samples of septic children and 32 whole blood samples of healthy children; GSE26378 included 82 whole blood samples of septic children and 21 whole blood samples of healthy children; GSE66099 included 229 whole blood samples of septic children and 47 whole blood samples of healthy children. The whole genome expression profiles of the above samples were detected by Affymetrix Human Genome U133 Plus 2.0 Array chip platform.

The age of the sample ranged from 0.1 to 9.8 years (see Supplementary Table 1 for detail). There was no significant clinical difference between the specimen of septic children and healthy children (gender, age, etc.).

The third International Consensus Definitions for Sepsis and Septic Shock (Sepsis 3) suggested the Sequential Organ Failure Assessment (SOFA) score to grade organ dysfunction in adult patients with suspected infection, which was not suitable for children illness. As reported previously, we used a pediatric version of the SOFA score (pSOFA) in this study (14).

The septic children in the study had a pSOFA score ≥2.

### Differential Gene Analysis

We firstly used the Robust Multi-Array Average (RMA) method to normalize the original data measured by the chip and then took the normalized value and log2 logarithm to generate the data after normalization, subsequently for differential expression analysis. We screened differentially expressed genes based on the limma function package of R language (version 3.5.2, the same below) (15). The absolute values of log-transformed differential expression multiple (Log2FC) >1 and FDR < 0.05 were used as a criteria.

### Functional Enrichment Analysis

For the obtained differentially expressed genes, we used the “clusterProfiler” function package in R language for enrichment analysis of GO (Gene Ontology, including Biological Process, Molecular Function, and Cellular Component) and KEGG (Kyoto Encyclopedia of Genes and Genomes, including key related pathways) analysis. When *p* < 0.05, we considered the corresponding entries to be significantly enriched (16).

### Protein–Protein Interaction Networks and Identification of Hub Genes

The STRING database is the one for analyzing and predicting protein functional associations and protein interactions. In this study, we utilized STRING (https://STRING-db.org/, version 11.0) to analyze protein functional associations and protein interactions (17). The Cytoscape (version 3.7.2) was used to visualize PPI network, and the cytoHubba plug-in in Cytoscape was used to screen the key genes (hub genes) in the PPI network based on the Algorithm of Maximum neighborhood component (MNC) (18).

### Calculation of Immune Infiltrating Cells

We used the software CIBERSORT (https://cibersort.stanford.edu) to calculate the relative proportions and *p*-values of 22 immune infiltrating cells in each sample. This software provided a pre-convolution algorithm to characterize the composition of immune invading cells based on the gene expression matrix using a deconvolution algorithm. CIBERSORT calculated the relative proportion of 22 immune infiltrating cells in each sample based on the expression of these 547 barcode genes as well as the *p*-value. The smaller the *p*-value, the higher the content of immune infiltrating cells in the sample.

### Logistic Regression Model Construction

Here, we used GSE26440 as a training set to construct the Logistic model, and GSE26378 and GSE66099 as two independent verification sets to verify the model. The part of the data set GSE66099 that coincided with GSE26440 and GSE26378 had been removed. The remaining 138 specimen were processed samples as independent verification sets in this study. Firstly, the samples were divided into two groups: normal control group and pediatric sepsis group. The GLM function in R language was used as a continuous variable, and the sample type was used as a binary response. A multifactor logistic regression model was constructed, and then the variables were further screened by stepwise regression. The model was then reconstructed using the screened variables and the *p*-value of each variable was calculated by the model. Finally, the candidate gene reconstruction model with *p* < 0.05 was selected as the final model for follow-up analysis.

The source code (in reproducible format) in this study has been shown in Supplementary Table 2.

### Construction of Random Forest Classification Model

In this study, the sample type was considered as a dependent variable, and the selected gene expression value was considered as an independent variable. The method of bootstrap sampling and the method of Bagging were utilized to generate multiple decision tree classifiers (implemented by the “randomForest” function package in R) and the final random forest model.

### ELISA Analysis of Hub Genes

A total of 63 (septic) children diagnosed by pathology and evaluated by SOFA score system in Tianjin People's Hospital from January 2018 to December 2020 were randomly selected and collected. Due to the limitation of sample size as well as in order to generate statistically convincing results, the patients were divided into three groups (control group: pSOFA = 0; mild pediatric sepsis group: pSOFA = 2–4; severe pediatric sepsis group: pSOFA score ≥ 5). There were 21 cases in each group. This study is in line with the medical ethics standards and approved by the hospital ethics committee. All treatment and testing were carried out after obtaining informed consent from patients or their families.

The concentrations of hub genes were determined by ELISA double antibody sandwich method from patients' peripheral blood. The specific operation was carried out in strict accordance with the instructions of the kit (Abcam company, US). The experimental results were repeated 3 times independently and were tested by statistical methods.

## Results

### Analysis Results of Differentially Expressed Genes

**Figure 1:**
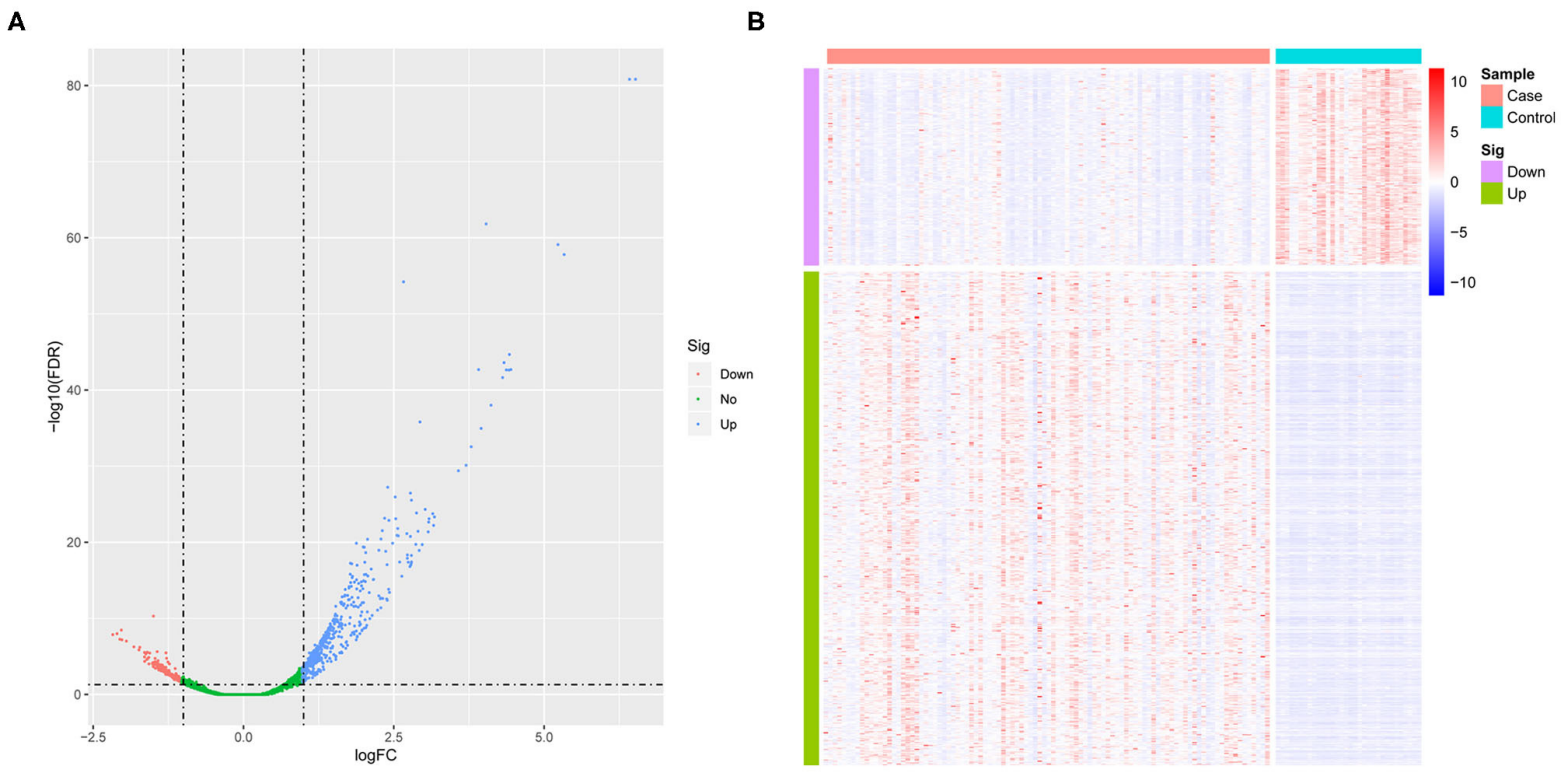
The analysis of differential gene. **(A)** The volcano map of differentially expressed genes. The horizontal axis represents the multiple of differential expression (Log2FC), and the vertical axis represents –log10 (FDR). Meanwhile, the blue dot indicates 507 up-regulated genes, and the red dot indicates 201 down-regulated genes. **(B)** The heat map of differentially expressed genes. The horizontal axis indicates sample, and the vertical axis indicates different genes; red color denotes high gene expression, and blue color indicates low gene expression.

We first standardized the microarray data of 3 sets of GEOs to remove batch effects. Using GSE26440 normalized data for differential gene analysis, we obtained a total of 708 differentially expressed genes in the pediatric sepsis group relative to the control group, including 507 up-regulated genes and 201 down-regulated genes (Figure 1A and Supplementary Table 3), and the expression of differentially expressed genes was significantly different between the disease group and the control group (Figure 1B).

### GO and KEGG Enrichment Analysis Results

**Figure 2:**
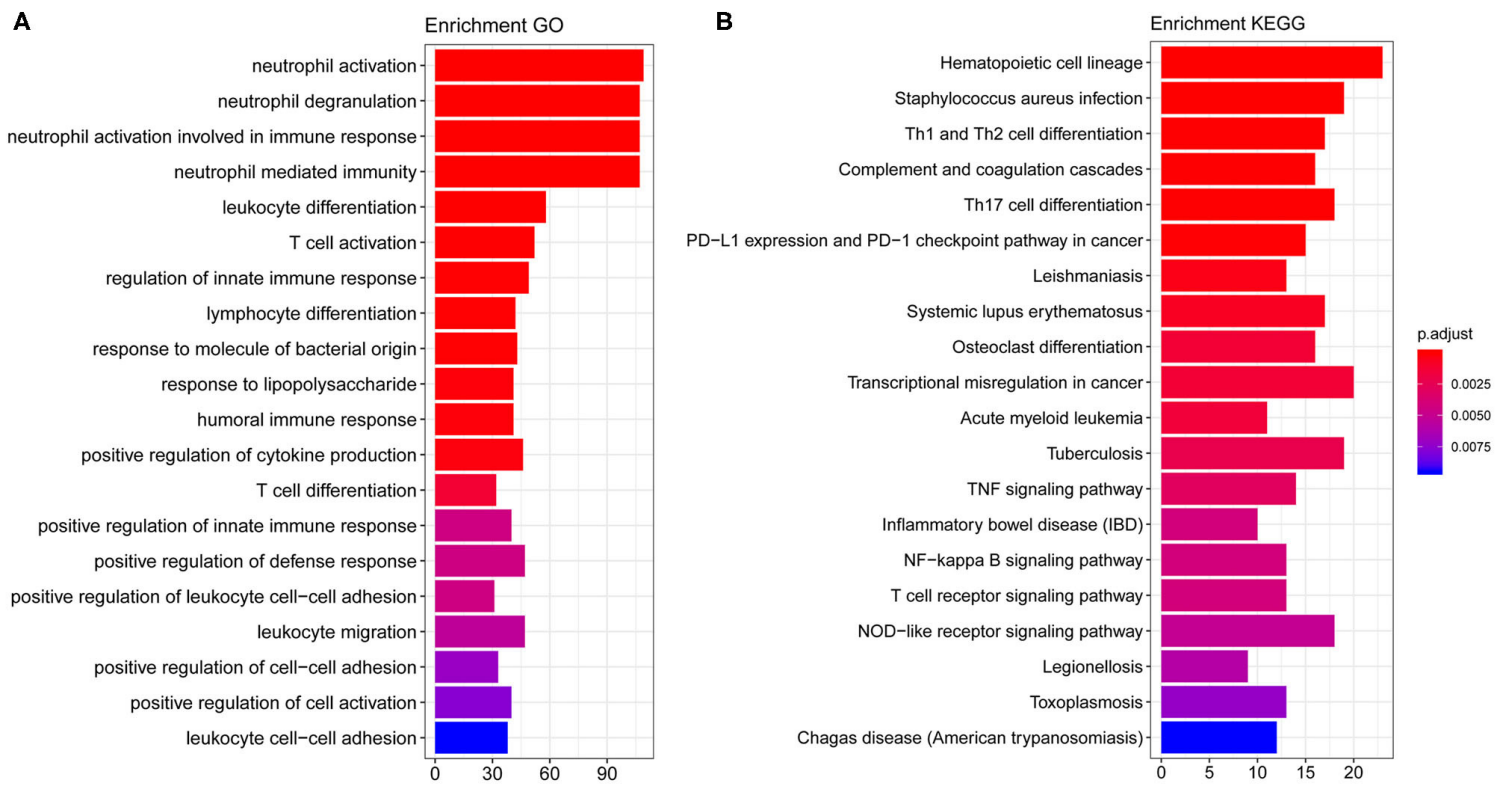
GO and KEGG enrichment results. **(A)** The top 20 GO term enrichment results with the largest number of genes. In the figure, the horizontal axis represents the number of enriched genes, and the vertical axis represents the name of each GO term. **(B)** The enrichment results of the 20 KEGG pathways with the largest number of genes. The horizontal axis in the figure indicates the number of genes enriched, and the vertical axis indicates the name of each KEGG pathway, respectively.

By performing GO and KEGG enrichment analysis on these 708 differentially expressed genes, we found that these 708 differentially expressed genes were enriched in GO terms related to immune cells such as neutrophil activation and neutrophil degranulation (Figure 2A). At the same time, the hematopoietic cell lineage and *Staphylococcus aureus* infection were significantly enriched in KEGG pathway analysis (Figure 2B).

### Immune Cell Calculation Results

**Figure 3:**
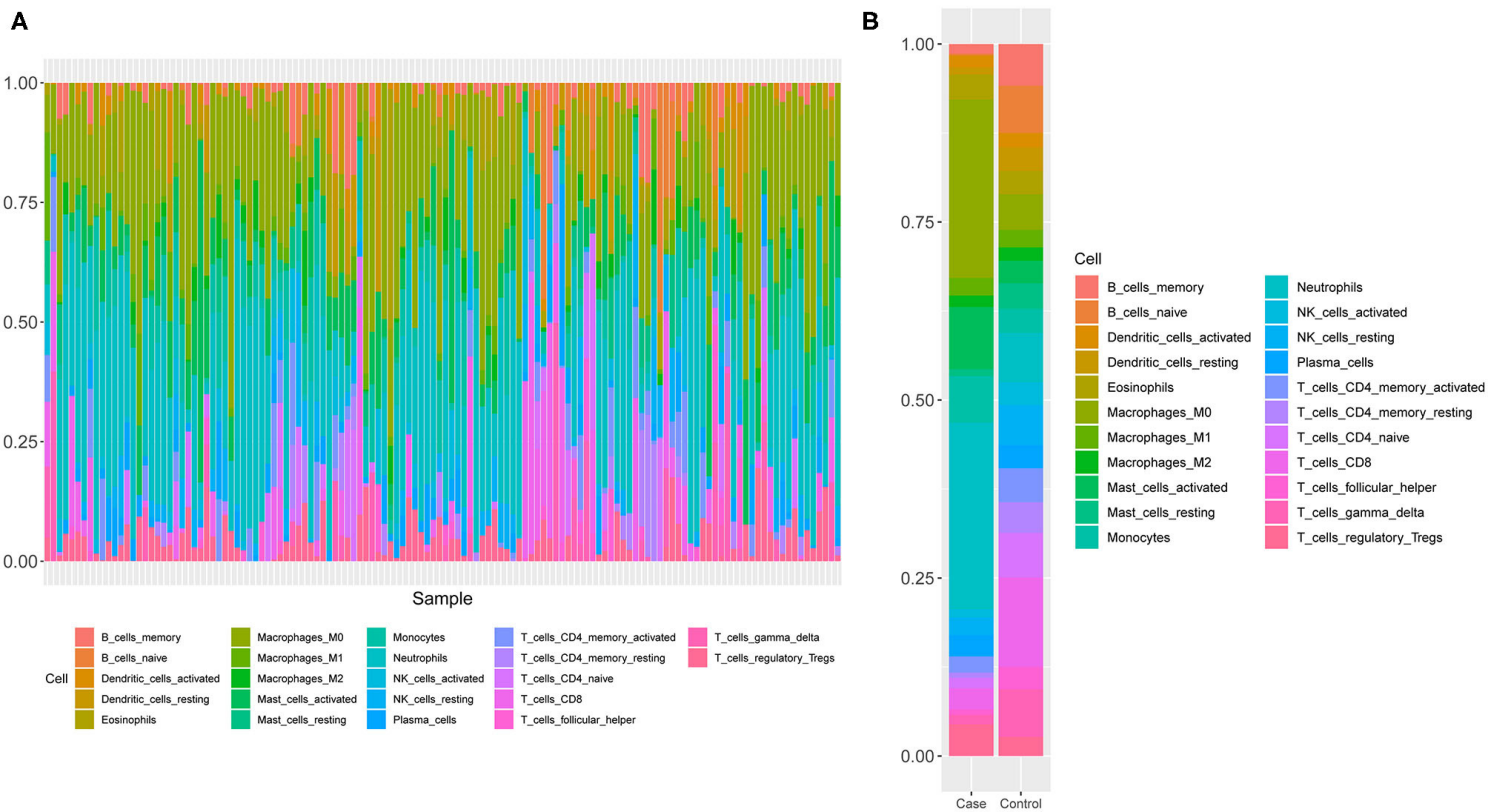
A schematic diagram of the difference between immune cells. **(A)** The composition of immune cells in the 130 samples. The horizontal axis represents 130 samples, and the vertical axis represents the percentage of each type of immune cells. **(B)** The composition of immune cells in two groups of samples. The horizontal axis indicates two groups of samples, and the vertical axis indicates the percentage of each type of immune cells.

Since GO and KEGG results showed the close correlation to immune cells, we next analyzed the immune cell composition in the samples to study the immunity in different groups of samples. Using the CIBERSORT algorithm, we explored the differences in immune infiltration among 22 immune cell subgroups for the 130 samples in GSE26440. We found that the relative proportions of 16 immune cells in 22 types of immune cells were significantly different between the two groups (Figure 3A), which included resting and activated memory CD4+ T-cells, follicular helper T-cells, T regulatory cells (Tregs), gamma/delta T-cells, activated NK cells, monocytes, macrophages, resting dendritic cells, resting, and activated mast cells, as well as neutrophils. Meanwhile, the proportion of neutrophils in the pediatric sepsis group was significantly higher than the normal group (Figure 3B).

### PPI Network Construction and Screening of Key Genes

**Figure 4:**
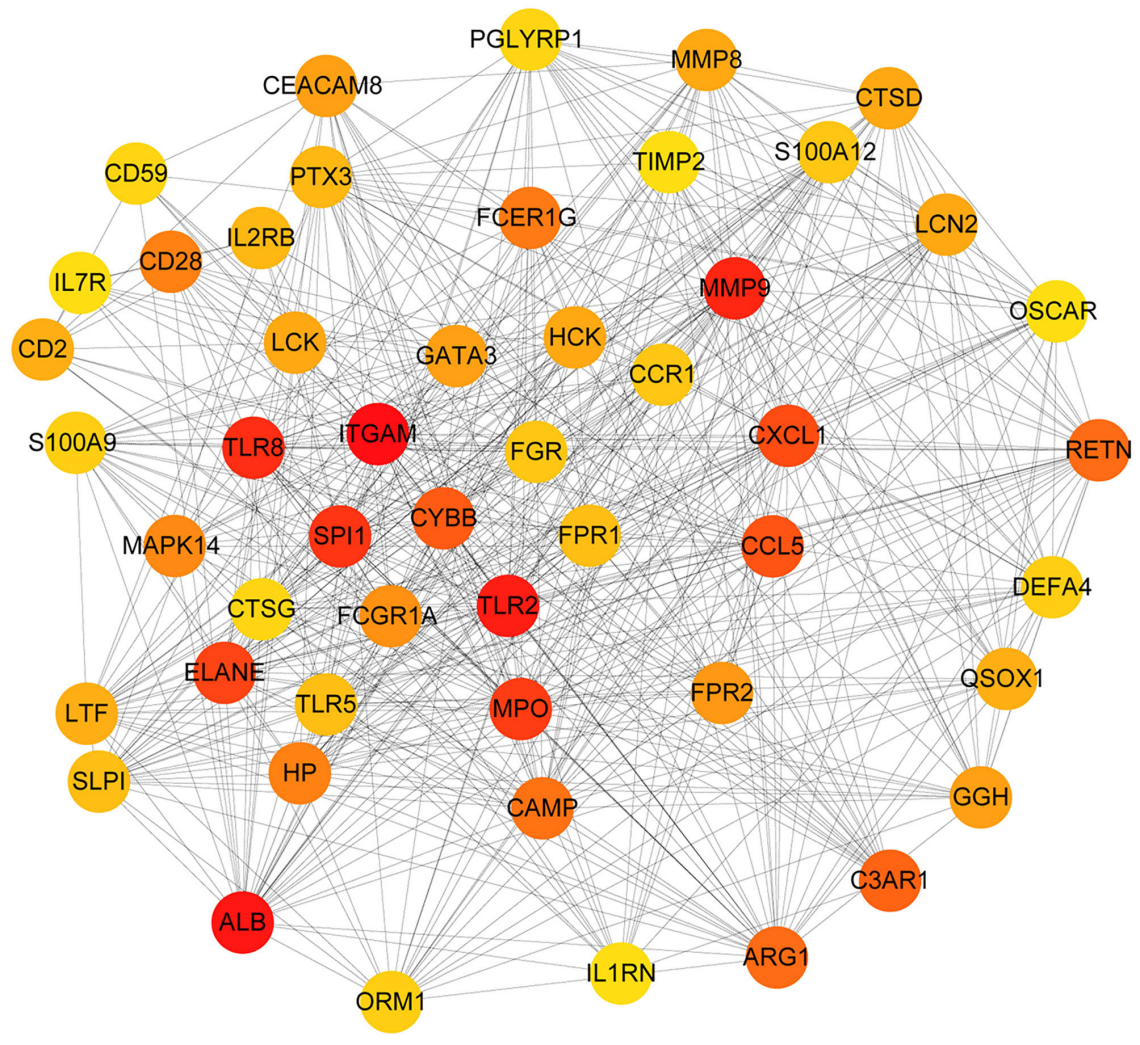
The PPI network diagram of key genes related to sepsis in children. Each dot represents a node. The darker the dot is, the more line segments are connected to the dot.

We established a PPI network of 708 genes by STRING database, as 577 genes with a confidence score ≥0.4. We used Cytoscape software and the MNC algorithm to score the importance of each node in the network. With these together, we developed the top 50 genes according to the score from large to small. The darker the color, the more important the node was (Figure 4).

### The Construction of the Logistic Model and the Random Forest Classification Model

**Figure 5:**
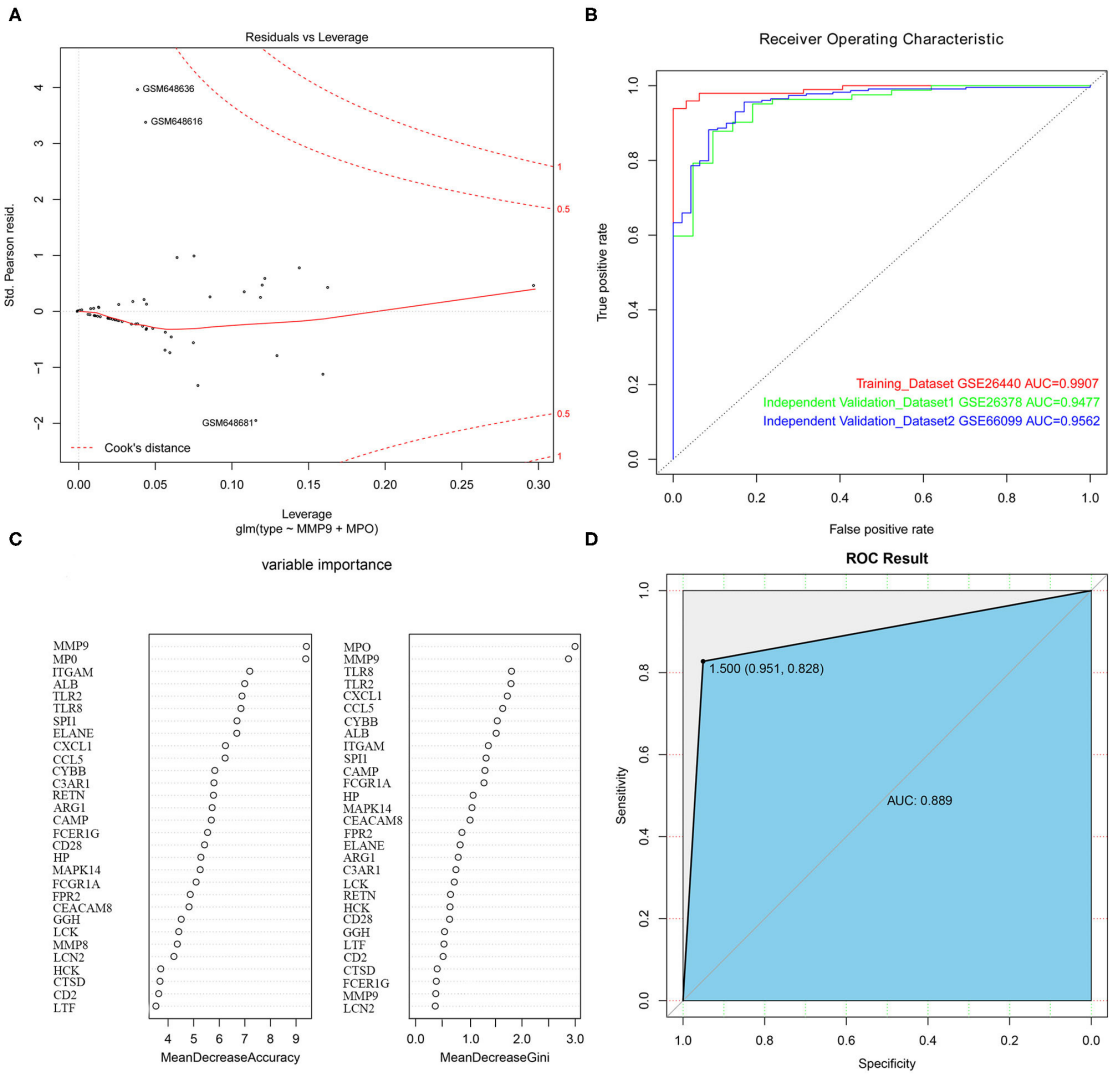
The construction of logistic regression prediction model and random forest classification model. **(A)** The Logistic model diagnostic chart. The red dashed line in the figure indicates the COOK distance. **(B)** The ROC curve. The horizontal axis represents the specificity of the FPR (false positive rate), and the vertical axis represents the sensitivity of the TPR (true positive rate). **(C)** The construction of the random forest classification model. **(D)** The ROC curve for the random forest classification model.

With the selected 50 genes, we generated logistic regression model 1 from the training set GSE26440. In order to use as few variables as possible to build a strong interpretation model, we performed a stepwise regression method to further identify 5 primary genes from these 50 genes, which were TLR2, MMP9, TLR8, MPO, and CCL5. Logistic regression model 2 was constructed by incorporating these 5 genes into the model as variables. An OR value >1 indicated that the expression of this factor was positively correlated with the onset of disease, while <1 was negatively correlated. At the same time, we found that the *p*-values of these 2 genes, MMP9 and MPO, were <0.05, indicating that they contribute greatly to the model while others (TLR2, TLR8, as well as CCL5 with *p*-value more than 0.05) were not used for model 3 construction and subsequent analysis. Furthermore, we reconstructed logistic regression model 3 with these 2 genes as the final model and found that this logistic regression model had no extreme point affecting the accuracy of the model. The red dashed line in the figure indicated the COOK distance. Generally, the point where the COOK distance >0.5 was a very “influential” point, which affected the reliability of the model. It could be seen in the figure that our model did not show such a point (Figure 5A). The AUC value in the training set GSE26440 was 0.9907, while the AUC values in GSE26378 and GSE66099 were 0.9477 and 0.9562, respectively (Figure 5B). The AUC as a numerical value can directly evaluate the quality of the model. The larger the value, the better the model.

To further evaluate the importance of the 2 hub genes, we also constructed a random forest classification model. The GSE26440 was used as training set. The sample type was used as a dependent variable. The expression of 50 genes selected in the previous step was used as an independent variable. See Figure 5C for details. The figure demonstrated the top 30 genes in the importance ranking of these 50 genes in the random variable model. The MeanDecreaseAccuracy indicated the decrease of model accuracy after variable replacement, while the MeanDecreaseGini indicated the decrease of model GINI coefficient after variable replacement. The larger the 2 values were, the more important the variable was. From the figure, it was clear that MMP9 and MPO were the top 2 genes in the MeanDecreaseAccuracy as well as MeanDecreaseGini's scores, indicating that these 2 genes were more crucial variables in the random forest model (Figure 5D).

The above results demonstrated that the model based on these 2 genes could be used as the primary criteria for pediatric sepsis staging.

### The Functional Validation of the Selected Biomarkers

To functionally verify the possibility of biomarkers for pediatric sepsis in clinic, we tested the concentrations of 2 target hub genes in pediatric sepsis patients' peripheral blood by ELISA method. As pediatric sepsis severity increases, the levels of MMP9 and MPO decreased significantly (*P* < 0.05, shown in Table 1).

**Table 1:** Selected gene concentrations comparison between different stages of pediatric sepsis patients.

	**Control group**	**Mild pediatric sepsis group**	**Severe pediatric sepsis group**
MMP9 (ng/mL, x ± s)	51.21 ± 13.14	23.28 ± 6.41	7.59 ± 2.33
MPO (ng/mL, x ± s)	76.32 ± 17.43	43.94 ± 10.38	18.62 ± 8.16

## Discussion

Sepsis can lead to death of children with a dramatic increase in the incidence of disease. The prominent problem for pediatric sepsis is the inflated misdiagnosis as well as the lack of a gold standard. Epidemiologic research has provided evidence that sepsis is more or less similar to over 50 systemic diseases in children (19–21). For instance, in the case of fever: immunosuppressed children do not always develop fever, so the infection is hard to detect. In contrast, critically ill children have a certain degree of hyperthermia but may not present infection (22). All of these may lead to the disease being ignored or misdiagnosed in the first place. Whenever symptoms are present, sepsis in children becomes more severe. All of these are all in dire need of effective forecasting targets or biomarkers for pediatric sepsis. To this end, it is beneficial to develop a comprehensive specific expression profile of genes in pediatric sepsis patients for potential candidates. Here, compared with control specimens, a total of 708 differentially expressed genes in pediatric sepsis were screened out, including 507 up-regulated genes and 201 down-regulated genes (see Figure 1A for details).

We further studied the biological process related to these genes using GO and KEGG enrichment analysis. We found that these target genes were significantly enriched in biological processes (BP) related to immune cells such as neutrophil activation, neutrophil degranulation, hematopoietic cell lineage, and *S. aureus* infection (see Figure 2 for details). Previously, a multicohort analysis by Sweeney et al. suggested that neutrophil activation, neutrophil degranulation, monocytes, as well as T cell-associated process had been involved in sepsis development and formation (23). They utilized data sets containing cohorts of children and adults, men and women, with a mix of community- and hospital-acquired sepsis, while we focused on pediatric sepsis; this may support the fact that these processes are common for all-age sepsis. This host response in septic progression involves many defense mechanisms with strong cellular activation, including neutrophil activation. In this process, neutrophil cells are key to innate immunity through their complex interactions with vascular cells, and their activation may be involved in systemic tissue damage. Their activation also leads to the release of neutrophil traps, which are involved in pathogen containment and phagocytosis, as well as coagulation activation (24). Several reports have demonstrated that neutrophils generally have a relatively high expression in sepsis patients (25, 26). Previously, a study using high-throughput technologies has been able to identify differentially expressed pediatric septic shock biomarkers using gene expression data to predict long-term outcomes (27). In this study, highly expressed genes in pediatric sepsis are enriched in multiple KEGG pathways and GO terms, which are related to neutrophils. Therefore, high expression of related genes may be one of the potential causes of increased neutrophil content in pediatric sepsis. On other hand, sepsis can be induced by viruses, bacteria, fungi, etc. The enrichment of neutrophils in the study might be due to the high infection rate of bacteria in children's specimen. Previously, it was suggested that sepsis was closely associated with hematopoietic stem cell exhaustion and hematopoietic cell lineage, which was processed through a Toll-like receptor 4 (TLR4)-related mechanism (28, 29). *S. aureus* is now the most common cause of bacteremia and infective endocarditis in industrialized nations worldwide and is associated with excess mortality when compared to other pathogens. It has been suggested that *S. aureus* is the primary cause of pediatric sepsis (30). Overall, these pathways are related to pediatric sepsis on some levels, and they provide a significant research starting point.

Due to complexity of pathogenesis in pediatric sepsis, it is impossible to study each potential single gene individually. To this end, an alternative method combining bioinformatics and machine learning is required for our study. Here, we used Cytoscape software and the MNC algorithm to identify the top 50 genes according to their scores in the model (see Figure 4B for details). Using the 50 genes, we generated a logistic regression prediction model. With further reconstruction, two primary genes including MMP9 and MPO had been screened out. Matrix metalloproteinases 9 (MMP-9) is a zinc-dependent gelatinase, which could decrease the expression of extracellular matrix proteins and influence the metastatic behavior of immune cells (31). MMPs are secreted as pro-MMPs, which are regulated by tissue inhibitors of metalloproteinase (TIMPs) as well as by α-macroglobulins (31). Previously, MMP-9, TIMP-1 levels, and MMP-9/TIMP-1 ratio have been suggested as biomarkers in adult severe sepsis and septic shock (32, 33). A study by Alqahtani et al. demonstrated that the MMP-9/TIMP-1 ratios can also serve as a biomarker for the identification of sepsis in pediatric patients (34). This is consistent with our integrated study. However, we did not find a significant difference for TIMP-1. This may be due to the fact that they used febrile controls in addition to a healthy control group as was often used in studies of biomarkers in sepsis, or the constant comparison in our report did not fluctuate time-dependent analysis as they performed. All of these deserve further investigation.

On the other hand, it is interesting to further explore the function of MPO in the pediatric sepsis study. The neutrophil myeloperoxidase (MPO) is mainly shown to promote oxidative stress by the production of active chlorinated molecules (35). It was rarely reported to be associated with pediatric sepsis, yet a small sample size study suggested a lower MPO level in pediatric sepsis compared with the control group (91.24 vs. 116.55 U/L; *p* value = 0.023) (36). These evidences highlighted the importance of understanding the relation between the MPO gene family pathway and pediatric sepsis, which initiated a tremendous starting point for the following study. Importantly, a recent study using two feature selection methods including Random Forest Feature Importance (RFFI) and Minimum Redundancy and Maximum Relevance (MRMR) also provided multiple differentially expressed genes and enriched pathways for pediatric sepsis. Within these, MPO was also a primary candidate (37). Using two potential target genes (MMP9 and MPO), we established a logistic regression model aiming for pediatric sepsis prediction. The accuracy of the model prediction was evaluated and approved by clinical data outcomes (Table 1), which demonstrated the tendency of two biomarkers' change for different levels of pediatric sepsis patients.

To conclude, in light of the fact that there remains no gold standard diagnosis and no reliable disease-specific prediction for pediatric sepsis, we summarized the differential expression profile of genes in the disease. Several target genes established a specific expression manner, which initiated new insights into the management of pediatric sepsis therapeutic biomarkers discovery and provided a very valuable data reference for future clinical research.

## Data Availability Statement

The original contributions presented in the study are included in the article/Supplementary Material, further inquiries can be directed to the corresponding author/s.

## Ethics Statement

The studies involving human participants were reviewed and approved by Tianjin Union Medical Center, Tianjin, China. Written informed consent to participate in this study was provided by the participants' legal guardian/next of kin.

## Author Contributions

All authors listed have made a substantial, direct and intellectual contribution to the work, and approved it for publication.

## Conflict of Interest

The authors declare that the research was conducted in the absence of any commercial or financial relationships that could be construed as a potential conflict of interest.

## Supplementary Material

Supplementary Table 1 The clinical information of sample specimen.

Supplementary Table 2 The source code of logistic regression model.

Supplementary Table 3 The results of differentially expressed genes.
