# Supplementary material for: Identification of Potential Diagnostic Gene Targets for Pediatric Sepsis Based on Bioinformatics and Machine Learning
Source: Front Pediatr. 2021 Mar 4;9:576585. doi: 10.3389/fped.2021.576585 (PMC7969637; doi:10.3389/fped.2021.576585)
Supplement: Supplementary Table 2 — The source code of logistic regression model. [file Table_2.docx]

The source code (in reproducible format)

Differentially expressed gene analysis

library("limma");

foldChange=1;

padj=0.05;

rawexprSet=read.csv("diff.csv",header=TRUE,row.names=1,check.names = FALSE);

group <- read.csv("sample.csv",header=TRUE,row.names=1,check.names = FALSE);

group <- group[,1];

design <- model.matrix(~0+factor(group));

colnames(design)=levels(factor(group));

rownames(design)=colnames(rawexprSet);

fit <- lmFit(rawexprSet,design);

cont.matrix<-makeContrasts(paste0(unique(group),collapse = "-"),levels = design);

fit2=contrasts.fit(fit,cont.matrix);

fit2 <- eBayes(fit2);

tempOutput = topTable(fit2,coef=1,n=Inf,adjust="BH");

nrDEG = na.omit(tempOutput);

allDiff <- nrDEG;

diff=allDiff;

write.csv(diff, "limmaOut.csv")

Functional enrichment analysis

a=read.csv("enrich.csv",header=T)

gene=as.character(a[,1])

gene=bitr(gene,fromType="SYMBOL",toType="ENTREZID",OrgDb="org.Hs.eg.db")

ego=enrichGO(gene=gene$ENTREZID,OrgDb='org.Hs.eg.db',ont="ALL",pAdjustMethod="BH",pvalueCutoff=0.5,qvalueCutoff=0.5,readable=TRUE)

GO=ego[1:10,c(1,2,3,9,7)]

GO$geneID=str_replace_all(GO$geneID,"/",",")

names(GO)=c("Category","ID","term","Genes","adj_pval")

circ <- circle_dat(GO,a)

chord <- chord_dat(data = circ, genes = a, process = GO$term)

GOChord(data=chord, title="",space = 0.01, gene.order = 'logFC', gene.space = 0.2, gene.size = 3,lfc.col=c('firebrick3','white','royalblue3'),process.label=8)

GOCircle(circ)

kk=enrichKEGG(gene=gene$ENTREZID,organism='hsa',keyType="kegg",pAdjustMethod="BH",pvalueCutoff=0.7,qvalueCutoff=0.7)

barplot(ego,showCategory=20,title="")

barplot(kk,showCategory=20,title="")

Logistic regression model

train=read.csv("log.csv",header=T,row.names=1)

model <- glm(type ~MMP9+MPO,family=binomial(link='logit'),data=train)

p <- predict(model, newdata=subset(train), type="response")

pr <- prediction(p, train$type)

auc <- performance(pr, measure = "auc")

auc <- auc@y.values[[1]]

test1=read.csv("log_test1.csv",header=T,row.names=1)

prf <- performance(pr, measure = "tpr", x.measure = "fpr")

p1 <- predict(model, newdata=subset(test1), type="response")

pr1 <- prediction(p1, test1$type)

auc1 <- performance(pr1, measure = "auc")

auc1 <- auc1@y.values[[1]]

prf1 <- performance(pr1, measure = "tpr", x.measure = "fpr")

test2=read.csv("log_test2.csv",header=T,row.names=1)

p2 <- predict(model, newdata=subset(test2), type="response")

pr2 <- prediction(p2, test2$type)

auc2 <- performance(pr2, measure = "auc")

auc2 <- auc2@y.values[[1]]

prf2 <- performance(pr2, measure = "tpr", x.measure = "fpr")

plot(prf,col="red",lwd=3)

abline(0,1,lty=3,lwd=1.5)

plot(prf1,add=T,col="blue",lwd=3)

plot(prf2,add=T,col="green",lwd=3)

text(0.75,0.2,col="red"," Training set GSE26440 AUC=0.9907")

text(0.62,0.15,col="blue","Independent Validation_Dataset1 GSE26378 AUC=0.9477")

text(0.58,0.10,col="green","Independent Validation_Dataset2 Part of GSE66099 AUC=0.9776")
